# Influence of Titanium Alloy Scaffolds on Enzymatic Defense against Oxidative Stress and Bone Marrow Cell Differentiation

**DOI:** 10.1155/2020/1708214

**Published:** 2020-07-29

**Authors:** Lais Morandini Rodrigues, Elis Andrade Lima Zutin, Elisa Mattias Sartori, Daniela Baccelli Silveira Mendonça, Gustavo Mendonça, Yasmin Rodarte Carvalho, Luana Marotta Reis de Vasconcellos

**Affiliations:** ^1^Department of Biosciences and Oral Diagnosis, São Paulo State University (UNESP), Institute of Science and Technology, São José dos Campos, Brazil; ^2^Department of Biological Sciences, Oakland University, Rochester Hills, MI, USA; ^3^Department of Oral Surgery and Integrated Clinics, São Paulo State University (UNESP), School of Dentistry, Araçatuba, Brazil; ^4^Department of Biological and Material Sciences & Prosthodontics, University of Michigan School of Dentistry, Ann Arbor, MI, USA

## Abstract

Studies have been directed towards the production of new titanium alloys, aiming for the replacement of Ti-6 Aluminium-4 Vanadium (TiAlV) alloy in the future. Many mechanisms related to biocompatibility and chemical characteristics have been studied in the field of implantology, but enzymatic defenses against oxidative stress remain underexplored. Bone marrow stromal cells have been explored as source of cells, which have the potential to differentiate into osteoblasts and therefore could be used as cells-based therapy. The objective of this study was to evaluate the activity of the antioxidant enzymes superoxide dismutase (SOD) and catalase (CAT) in porous scaffolds of Ti-6 Aluminium-4 Vanadium (TiAlV), Ti-35 Niobium (TiNb), and Ti-35 Niobium-7 Zirconium-5 Tantalum (TiNbZrTa) on mouse bone marrow stromal cells. Porous titanium alloy scaffolds were prepared by powder metallurgy. After 24 hours, cells plated on the scaffolds were analyzed by scanning electron microscopy (SEM). The antioxidant enzyme activity was measured 72 hours after cell plating. Quantitative real time PCR (qRT-PCR) was performed after 3, 7, and 14 days, and *Runx2* (Runt-related transcription factor2) expression was evaluated. The SEM images showed the presence of interconnected pores and growth, adhesion, and cell spreading in the 3 scaffolds. Although differences were noted for SOD and CAT activity for all scaffolds analyzed, no statistical differences were observed (*p* > 0.05). The osteogenic gene *Runx2* presented high expression levels for TiNbZrTa at day 7, compared to the control group (TiAlV day 3). At day 14, all scaffolds had more than 2-fold induction for *Runx2* mRNA levels, with statistically significant differences compared to the control group. Even though we were not able to confirm statistically significant differences to justify the replacement of TiAlV regarding antioxidant enzymes, TiNbZrTa was able to induce faster bone formation at early time points, making it a good choice for biomedical and tissue bioengineering applications.

## 1. Introduction

For a biomaterial to be considered excellent for bone replacement, it must show characteristics that are compatible with its use in the long term, that there are no adverse tissue reactions, that it has excellent corrosion resistance in the body, and that it exhibits high mechanical strength and fatigue resistance, low modulus of elasticity, and good wear resistance [1, 2]. Titanium and Ti6Al4V alloy have been indicated as the best biomaterial for bone replacement due to their excellent physical and biocompatibility properties [3, 4]. However, one of its main disadvantages is the large difference in the elasticity modulus between biomaterial and bone [5–7]. This is particularly important because it can lead to long-term bone loss and consequent implant failure. TiAlV alloy, widely used in the field of orthopedics, exhibits another disadvantage: its aluminum (Al) and vanadium (V) components have cytotoxic potential and can be released in the long term, causing adverse biological effects [8]. Therefore, studies have been directed towards the production of new titanium alloys using other metals, aiming to improve their characteristics, especially with respect to the modulus of elasticity and long-term cytotoxicity [9, 10]. There are several studies showing the effectiveness of titanium bonding to metals such as niobium (Nb), zirconium (Zr), and tantalum (Ta) resulting in noncytotoxic alloys, with good biocompatibility and modulus of elasticity closer to the bone [11–15]. Porosity is another feature of titanium alloys that has been extensively studied in order to improve stress shielding in the bone-implant interface due to mismatching of elastic modulus between implant and bone [16–18]. Studies show that interconnected pores increase bone to implant contact and can be more favorable for transport and exchange of substances necessary for cell growth [16–19], allowing bone growth within the biomaterial, leading to a good biological fixation [20]. The creation of interconnected pores may also help decrease the modulus of elasticity, which would reduce the shielding stress, thus extending the implant life [21, 22]. Porous titanium alloys have been studied for their physical and chemical characteristics, biocompatibility, and osteogenesis potential, but there are no studies evaluating oxidative stress on these biomaterials when they are in contact with cells or the role of oxidative stress in the failure of biomaterials in the long term. Oxidative stress occurs when there is a difficulty or lack of elimination of reactive oxygen species, which in high doses can cause damage and irreversible injury to the cells and consequently to the tissues [23–25]. There are defense mechanisms against oxidative stress, called primary antioxidant enzymes such as CAT and SOD. These are able to neutralize reactive oxygen species (ROS), making them harmless to the cellular environment [26].

Bone marrow stromal cells have been widely used in studies testing biomaterials biocompatibility [27–30]. These cells are considered good candidates for bone tissue engineering and cells-based therapy because they can be easily obtained, are a good source of stem cells, have strong proliferative capacity, and have the potential to differentiate into different cell types, including osteoblasts [31]. An important early osteogenic marker is called *Runx2.* This gene is essential for mesenchymal stem cells differentiation into the osteoprogenitor lineage, and its presence excludes the possibility of these cells becoming adipocytes or chondrocytes [32].

Our study hypothesis was that implants composition can stimulate the production of ROS, generating extra activity of antioxidants enzymes SOD and CAT, which could alter cells response ultimately resulting in implant loss. Therefore, we analyzed SOD and CAT activity on TiAlV, TiNb, and TiNbZrTa porous scaffolds alloys on mouse bone marrow stromal cells. Also, we explored how these cells would respond to the oxidative stress caused by chemical composition of these biomaterials, in terms of cell attachment and spreading, as well as their ability to differentiate into osteoblasts by analyzing mRNA levels of osteogenic gene *Runx2*. We believe that our results can contribute in different ways for a possible substitution of TiAlV alloy, which is one of the major current challenges in implantology.

## 2. Materials and Methods

### 2.1. Porous Scaffold Preparation

Porous scaffolds of TiAlV (control group), TiNb (test group), and TiNbZrTa (test group) in this study were previously prepared by our research group using a special powder metallurgy process described in Vasconcellos et al. [33]. The powder of pure Ti grade II (CpTi) (purity 99.5%, <8 *μ*m) was obtained at the Department of Science and Aerospace Technology (DCTA) Materials Division of the Institute of Aeronautics and Space (AMR/IAE). It was mixed with the powders aluminum (purity 99.5%, <5 *μ*m), vanadium (99.9%, <325 mesh), zirconium (purity 99.5%, <325 mesh), tantalum (purity 99.9%, <325 mesh), and niobium (purity 99.8%, <45 *μ*m) (Sigma-Aldrich, St. Louis, MO, USA). In order to create porosity, alloy powders were mixed with urea (purity > 98.0%, powder) (Sigma-Aldrich, St. Louis, MO, USA), which acted as a spacer. To remove the urea, the samples were incubated in a vacuum oven (Marconi, Piracicaba, São Paulo, Brazil), at 200°C for 2 hours. A total of 48 samples were made in disc-shaped scaffolds, fabricated using a steel mold: 12 mm in diameter and 5 mm in height. The porosity of the samples was 40% and the pores showed a mean diameter of 300 *µ*m. The samples were carefully grouped, since it was not possible to see differences macroscopically. The nomenclature TiAlV, TiNb, and TiNbZrTa will be used throughout the text for each one of the groups.

### 2.2. Cell Culture

The project was approved by the Institutional Animal Care and Use Committee (IACUC) at the University of Michigan and is in accordance with the ARRIVE guidelines. Bone marrow stromal cells from femurs of male mice Tg (Sp7/mCherry) 2Pmay/J were collected. Two male mice (average body weight of 30 grams and 7-8 weeks old) were used for this purpose. The animals were housed in individual cages in an air-conditioned room, with freely available water and food and an artificial day/night cycle of 12 hours/12 hours. Euthanasia was performed by carbon dioxide inhalation, and femurs were removed to be processed for experiments. Femurs were dissected, and the proximal epiphysis were cut off so that the bone marrow could be flushed out. The femurs were placed in a 200 *μ*l pipette tip with cut ends, placed in a 1.5 ml microcentrifuge tube, and centrifuged at 2,000 rpm for 5 minutes in order to obtain the bone marrow cells. They were resuspended in 1 ml of MEM-Alfa modified with Earle's Salts (MEM-*α*) (Gibco-Life, Grand Island, NY, USA) growth media supplemented with 10% fetal bovine serum (Gibco) and antibiotic/antimycotic (penicillin/streptomycin/amphotericin B) (Sigma Chemical Co., St. Louis, MO, USA). Red blood cells were lysed using ammonium chloride solution according to the manufacturer's protocol (Stemcell Technologies, Vancouver, BC, Canada). The total number of cells was counted with a hemocytometer, and 1.5 × 10^6^ cells were plated onto prepared titanium scaffold alloys in 250 *μ*l of growth media detailed above.

### 2.3. Sample Characterization

One sample of each scaffold was examined under a high-resolution scanning electron microscope (Philips XL30 FEG, SEM, Philips, Eindhoven, Netherlands) after 24 hours of cell culture. The samples were washed two times with PBS and fixed with paraformaldehyde 10% for one hour. Paraformaldehyde was removed and the samples were washed 3 times with PBS. Concentrations of ethanol starting at 50% until 100% were added sequentially, for 10 minutes each. Ethanol was removed from samples, and after the samples dried, they were covered by gold. Observations were made at three randomly selected points on the titanium alloy scaffold, at four different magnifications.

### 2.4. Superoxide Dismutase Activity

For SOD activity, 3 scaffolds of each group were used. Cells were harvested from the scaffolds 72 hours after seeding. The activity of antioxidant enzyme SOD was measured using a Superoxide Dismutase Assay Kit (Cayman Chemicals, Ann Arbor, MI, USA). SOD activity utilizes a tetrazolium salt for detection of superoxide radicals generated by xanthine oxidase and hypoxanthine. The unit U/mg protein is defined as amount of enzyme needed to exhibit 50% of dismutation of the superoxide radical. The assay used measured total SOD activity, and the absorbance was set to 450 nm. Media were removed, and 250 *µ*l of trypsin-EDTA with a concentration of 0.05 ml/mg (Gibco) was placed on scaffolds. This step was necessary due to the fact that since the scaffolds were porous, cells had grown and adhered inside the scaffolds. After one minute, cells were centrifuged at 2,000 rpm for 10 minutes at 4°C in order to remove the trypsin-EDTA. The cell pellets were then homogenized in cold 20 mM HEPPES buffer, pH 7.2, containing 1 mM EGTA (ethylene glycol-bis (*β*-aminoethyl ether)-N,N,N′,N′-tetraacetic acid), 210 mM manitol, and 70 mM sucrose. This mixture was centrifuged at 1,500 rpm for 5 minutes at 4°C. The supernatant was collected and placed on ice for analysis. Protein was quantified using protein assay kit (Precision Red Advanced Protein Assay, Cytoskeleton) with Biotek Nova spectrophotometer at 595 nm wavelength (PowerWave HT). Experiments were performed in triplicate following the manufacturer's instructions.

### 2.5. Catalase Activity

For CAT activity, 3 scaffolds of each group were used. Seventy-two hours after plating, cells were collected from the scaffolds and the activity of the antioxidant enzyme CAT was measured using a Catalase Assay Kit (Cayman Chemicals, Ann Arbor, MI, USA). This kit utilizes the peroxidatic function of CAT for determination of enzyme activity in nmol/min/mg protein. This method is based on the reaction of the enzyme with methanol in the presence of an optimal concentration of H_2_O_2._ Absorbance is read at 540 nm. Media were removed, and 250 *µ*l of trypsin-EDTA at a concentration of 0.05 ml/mg (Gibco) was placed on the scaffolds. After one minute, the cells were centrifuged at 2,000 rpm for 10 minutes at 4°C in order to remove the trypsin-EDTA. The cell pellets were then homogenized on ice in 1-2 ml of cold buffer 50 mM potassium phosphate, pH 7.0, containing 1 mM EDTA. The mixture was centrifuged at 10,000 rpm for 15 minutes at 4°C. The supernatant was removed and placed on ice for analysis. Protein was quantified as described above. Experiments were performed in triplicate following the manufacturer's instructions.

### 2.6. RNA Isolation and *Runx2* Analysis

Experiment was performed in triplicate, and a total of 9 scaffolds of each group were used. Cells were collected 3, 7, and 14 days after plating. Media were removed, and scaffolds were rinsed twice with cold phosphate-buffered saline. For evaluation of mRNA expression on the titanium alloy scaffolds, adherent cells in each sample were lysed using TRIzol (Invitrogen, Carlsbad, CA). Cell lysates were collected by pipetting and centrifugation. Samples were kept frozen at 80°C for at least 24 hours. Total RNA in the cell lysates was collected by ethanol precipitation, according to the manufacturer's protocol. Total RNA was quantified using a spectrophotometer (PowerWave HT-BioTek Instruments) and the Gen5™ program (BioTek Instruments). From each total RNA sample, cDNA was generated using SuperScript VILO cDNA Synthesis (Invitrogen) in a standard 20 *µ*l reaction using 50 ng of the total RNA. Subsequently, equal volumes of cDNA were used to program qPCR reactions specific for mRNAs encoding the early osteogenic marker *Runx2* (Qiagen, Germantown, MD). The qRT-PCR reaction was performed in an Applied Biosystems 7900HT Real Time PCR System (Thermo Fisher Scientific, Waltham, MA, USA). Relative mRNA abundance was determined by the 2^−ΔΔCt^ method and reported as a fold change. TiAlV scaffold at day 3 was used as control group. *GAPDH* abundance was used for normalization.

### 2.7. Statistical Analysis

GraphPad Prism 8 (GraphPad, San Diego, CA) was used for statistical analyses. Data were analyzed using one-way ANOVA, followed by Tukey's test when necessary. The t-test was used for comparison between TiAlV control group and the other test groups for qRT-PCR analysis. The level of significance was set at *p* < 0.05 for all statistical analyses.

## 3. Results

### 3.1. Surface Analysis

**Figure 1:**
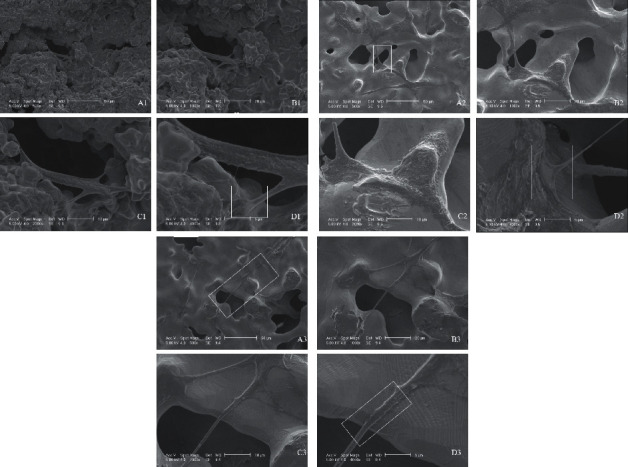
Scanning electron microscopy of mouse bone marrow stromal cells growing on TiAlV, TiNb, and TiNbZrTa scaffolds. 1.5 × 10^6^ cells were plated on each scaffold. After 24 hours, cells were prepared for SEM. It is possible to see the presence of interconnected pores and cell interaction at 500x magnification for the alloys TiAlV (A1), TiNb (A2), and TiNbZrTa (A3). Cells are spreading at 1000x (B1, B2, and B3) and 2000x magnification (C1, C2, and C3) for TiAlV, TiNb, and TiNbZrTa, respectively. There are clear cell adhesion and increased filopodia at a higher magnification of 4000x (D1, D2, and D3) for all alloys. Scale bar: (A1, A2, and A3) 50 *µ*m, (B1, B2, and B3) 20 *µ*m, (C1, C2, and C3) 10 *µ*m, and (D1, D2, and D3) 5 *µ*m.

At low magnification (500x), SEM images showed that all titanium alloy scaffolds had the presence of interconnected pores (Figure 1 (A1, A2, and A3)). At this resolution, it was possible to see the presence of cells interacting with the porous created in the scaffolds. At resolutions of 1000x and 2000x (Figure 1 (B1, B2, and B3 and C1, C2, and C3)), SEMs revealed growth and spreading of cells in all scaffolds. At a higher magnification of 4000x (Figure 1 (D1, D2, and D3)) it was possible to see better the interaction between the cells, with excellent adhesion, cell spreading, and presence of filopodia and lamellipodia.

### 3.2. Superoxide Dismutase Activity

**Figure 2:**
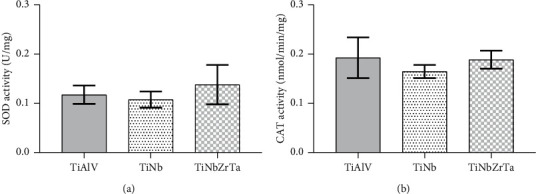
Superoxide dismutase (SOD) activity (a) in bone marrow stromal cells cultured in titanium scaffold alloys. The data are presented as mean ± standard deviation. No statistically significant differences were observed between the groups (*p* < 0.05). TiAlV, Ti-6 Aluminium-4 Vanadium; TiNb, Ti-35 Niobium; TiNbZrTa, Ti-35 Niobium-7 Zirconium-5 Tantalum. (b) Catalase activity in bone marrow stromal cells cultured in titanium scaffold alloys. The data are presented as mean ± standard deviation. No statistically significant differences were observed between the groups (*p* < 0.05). TiAlV, Ti-6 Aluminium-4 Vanadium; TiNb, Ti-35 Niobium; TiNbZrTa, Ti-35 Niobium-7 Zirconium-5 Tantalum.

A slight SOD activity increase was observed for the test group TiNbZrTa scaffold. For TiAlV and TiNb scaffolds, SOD activity was similar, but TiNb showed less activity for this antioxidant enzyme. However, no statistically significant differences were observed (Figure 2(a)).

### 3.3. Catalase Activity

Similar results of CAT activity were observed for TiAlV and TiNbZrTa (Figure 2(b)). TiNb scaffold showed the lowest activity for this antioxidant enzyme, compared to the other samples. However, no statistically significant differences were observed between the groups (*p* < 0.05).

### 3.4. *Runx2* Expression (qRT-PCR)

**Figure 3:**
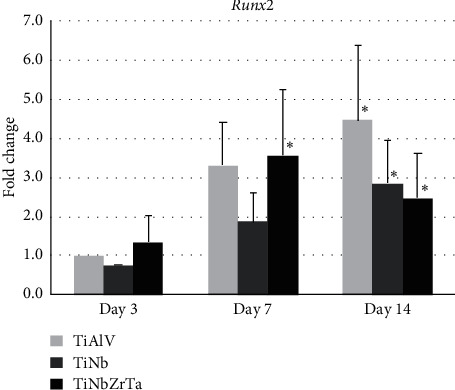
Time-dependent expression of Runt-related transcription factor2 (Runx2) gene in bone marrow stromal cells cultured in titanium scaffold alloys. The results are shown as fold change (2-ΔΔCt method, baseline = TiAlV day 3. ^*∗*^Statistically significant difference when compared with baseline (*p* < 0.05). TiAlV, Ti-6 Aluminium-4 Vanadium; TiNb, Ti-35 Niobium; TiNbZrTa, Ti-35 Niobium-7 Zirconium-5 Tantalum.

The results presented show the relative levels of *Runx2* mRNA expression compared to the control group: TiAlV scaffold at day 3. Our results showed a continuous increase of *Runx2* mRNA levels for TiNb alloy, reaching 1.8-fold upregulation after day 7. The same trend was observed for the control alloy TiAlV, which showed a fold induction of 3.3 and 4.4 after 7 and 14 days of cell culture, respectively. For the alloy TiNbZrTa, an upregulation of 3.5-fold was observed at day 7 and an upregulation of 2.4-fold was observed at day 14. Statistically significant differences were observed for TiNbZrTa at day 7 and between all groups after 14 days of cell culture (Figure 3).

## 4. Discussion

The chemical composition of the titanium alloys, as well as their topographic structural characteristics, have been the focus of studies aiming to improve not only the modulus of elasticity of these implants but also to promote good osseointegration and avoid future problems [14, 34], such as aseptic loss of implant, which is a complication of unknown etiology [23]. Therefore, there is a need to investigate these new titanium alloys, not only with tests that show their biocompatibility and absence of cytotoxicity, but also to evaluate other cellular mechanisms that may be linked to implant loss, since it is believed that the osseointegration process cellular response may be closely related to possible long-term failure [35].

The SEM analysis showed some evidence that the cells were in the process of cell differentiation because they presented shape change. The images from the electron scanning microscopy also showed the topographic characteristics of the samples, with the presence of interconnected pores that mimic the porous structure of bone. Some authors suggest that surfaces that mimic the innate characteristics of bone could improve bone cell response by mimicking the bone cellular environment [36, 37]. Modification of the material topography is the most important criteria for the production of biomaterials, since it is believed to be decisive for cell-material interaction and for the integration of the material with the surrounding tissue [38–40]. Hosseini et al. [41] believe that 3D scaffolds solve the problem with the blood supply, which can go toward the spaces, taking important molecules necessary for the healing process. Furthermore, according to Enderami et al. [42], interconnected pores in three dimensions serve as the temporary extracellular cell matrix for cell adhesion, growth, and differentiation. This was exactly what we observed in all porous scaffolds, where there was a large number of cells growing and adhering to the scaffolds only 24 hours after cell seeding. This fact is extremely important and desirable in the osseointegration process, since clinical success of the implants is related to rapid osseointegration [43], thus decreasing the chances of failure due to bacteria and other etiological agents that may be related to implant failure. We attributed the success of cell adhesion and spreading to the interconnected pores present on the scaffold biomaterial which allowed greater anchoring of the cells. Although 3D topographic features and the presence of porous increased the contact surface between cells and scaffolds, the main limitation of this study was to remove the cells from this biomaterial. Since the titanium alloy surfaces were completely porous, we could not scrape the cells off of them. In order to obtain the maximum number of cells in contact with the surface of the biomaterial, we used trypsin in low concentration and centrifuged the scaffolds.

The dysregulation of SOD and CAT can disturb inflammatory processes and contribute to implant loss, as affirmed by Tsaryk et al. [24]. Our hypothesis was based on the fact that TiAlV and its cytotoxic characteristic could generate extra oxidative stress and an unbalance of antioxidant enzymes, which could lead to implant loss. Some authors suggest replacing TiAlV for TiNb or TiNbZrTa based on evidences showing absence of cytotoxicity, good machinability, and mechanical strength of these alloys [12, 42–47], but do not test the stress caused by these alloys. Since there are no statistically significant differences between the scaffolds, only with SOD and CAT activity results our hypothesis around TiAlV could not be confirmed. We believe that all elements used in the composition of the biomaterials studied here had similar influence on cell response against oxidative stress.

This study used bone marrow stromal cells from mouse femur, since our objective was also to evaluate if these cells can differentiate into osteoprogenitor cells and how much the chemical composition associated with surface topography would induce this differentiation. Thus, we evaluated the expression of *Runx2*, a key transcription factor involved in the process of cell differentiation; its presence indicates that bone marrow stromal cells are differentiating into preosteoblasts and osteoblasts which, when mature, will secrete bone matrix [7, 48]. Even though it was difficult to scrape cells off from the porous scaffolds as mentioned before, TRIzol was sufficient to lyse the cells for RNA isolation and subsequent qRT-PCR. Our results showed that TiNb scaffold presented the lowest *Runx2* expression levels at earlier time points. The scaffold TiNbZrTa induced higher *Runx2* mRNA levels at earlier time points which indicates rapid osteoblastic differentiation for this sample and the lowest fold change after 14 days. Due to the fact that *Runx2* was expressed at the beginning of the differentiation process, small changes in the level of expression were already sufficient to trigger the osteogenesis process. It is also important to note that *Runx2* elevations are related to an increase in the expression of other bone-related genes such as alkaline phosphatase, collagen type I, osteocalcin, and osteopontin [48, 49], genes closely linked to the process of bone formation. Therefore, even though we have not analyzed any of the aforementioned genes, we believe that TiNbZrTa porous scaffold can improve the osseointegration process.

## 5. Conclusion

In our study, we were able to observe the presence of cell growth, adhesion, and spreading in all scaffolds analyzed, concluding that these scaffolds presented good chemical and topographic characteristics for promoting osseointegration. The analysis of the SOD and CAT activity did not show differences between the alloys. However, the osteogenic marker *Runx2* in the quaternary alloy TiNbZrTa demonstrated a better capacity for bone marrow stromal cell to differentiate into osteoblasts, which can improve the osseointegration process and could be a good choice for biomedical applications.

## Data Availability

The data used to support the findings of this study are available from the corresponding author upon request.
